# Assessing the Role of Past Depression in Patients with Mild Cognitive Impairment, with and without Biomarkers for Alzheimer’s Disease

**DOI:** 10.3233/JAD-221097

**Published:** 2023-04-18

**Authors:** Angela C. Golas, Patrick Salwierz, Tarek K. Rajji, Christopher R. Bowie, Meryl A. Butters, Corinne E. Fischer, Alastair J. Flint, Nathan Herrmann, Linda Mah, Benoit H. Mulsant, Bruce G. Pollock, Foad Taghdiri, Wei Wang, M. Carmela Tartaglia, on behalf of the PACt-MD Study Group

**Affiliations:** a Centre for Addiction and Mental Health, Toronto, ON, Canada; b Department of Psychiatry, Temerty Faculty of Medicine, University of Toronto, Toronto, ON, Canada; c Department of Psychology, Queen’s University, Kingston, ON, Canada; d Department of Psychiatry, University of Pittsburgh, Pittsburgh, PA, USA; e Keenan Research Centre for Biomedical Science, St. Michael’s Hospital, Toronto, ON, Canada; f University Health Network, Toronto, ON, Canada; g Sunnybrook Health Sciences Centre, Toronto, ON, Canada; h Rotman Research Institute, Baycrest Health Sciences, Toronto, ON, Canada; i Tanz Centre for Research in Neurodegenerative Diseases, University of Toronto, Toronto, ON, Canada; j Division of Neurology, Department of Medicine, University of Toronto, Toronto, ON, Canada; k Toronto Dementia Research Alliance, University of Toronto, Toronto, ON, Canada

**Keywords:** Alzheimer’s disease, cerebrovascular disease, major depressive disorder, mild neurocognitive disorder

## Abstract

Major depressive disorder (MDD) is a risk factor for Alzheimer’s disease (AD). Cerebrovascular disease (CVD) is implicated in MDD and AD. Our study compared participants with AD positive and negative cerebrospinal fluid (CSF) biomarkers on neuropsychological performance, remitted MDD status, and CVD burden. Next, we compared AD-CSF biomarkers and white matter hyperintensities (WMH) burden among three groups: mild cognitive impairment (MCI) (*n* = 12), MCI with remitted MDD (MDD+MCI) (*n* = 12), and remitted MDD alone (MDD) (*n* = 7). Few participants (18%) with MCI+MDD exhibited AD(+) biomarkers. Nearly all participants had moderate-severe WMH. WMH may contribute to cognitive impairment or depression in MCI patients with AD(-) biomarkers.

## INTRODUCTION

A diagnosis of major depressive disorder (MDD) increases the risk of dementia by two-fold or greater [1]. Furthermore, depression has been shown to accelerate progression from mild cognitive impairment (MCI), referencing an intermediate stage of cognitive impairment between normal cognitive aging and dementia, to dementia [2]. This finding has been replicated consistently in late-onset depression (LOD), with mixed results in early-onset depression (EOD) [3–5]. It has been posited that EOD and LOD represent distinct phenotypes and pathological processes. Early adverse events and genetic predispositions are implicated in EOD, while accumulation of vascular burden and neuropathological substrates contribute to LOD [6–8].

Late-life depression (LLD), which includes EOD that reoccurs in late-life, is associated with deficits in verbal and visuo-spatial memory, executive function, and processing speed [9, 10]. Furthermore, these cognitive deficits commonly persist after remission of depressive symptoms and appear to increase the risk of dementia through poorly understood neuropathological processes [11–13]. Inflammation and increased glucocorticoid production, which can contribute to amyloid deposition, accelerated hippocampal atrophy, and cerebrovascular disease (CVD) are implicated in LLD [6, 14]. There is conflicting evidence to support a direct link between amyloid-β (Aβ) and tau, two of the pathological hallmarks of Alzheimer’s disease (AD) and LLD [15–24]. Furthermore, many of these studies involve actively depressed individuals, with a paucity of research addressing the association of remitted depression and AD pathology [25].

CVD, as represented by white matter hyperintensities (WMH) on T2-weighted structural magnetic resonance imaging (MRI), is a common co-pathology of neurodegenerative diseases, particularly AD [26, 27]. White matter hyperintensities and AD pathology appear to have independent and additive effects on dementia risk [28, 29]. Among patients with normal cognition [30–32], MCI [30], and AD dementia [28–36], WMH are associated with lower levels of cerebrospinal fluid (CSF) Aβ_42_ [28]. WMHs are also associated with depression [7, 36]. The link between depression and CVD appears to be bidirectional: depression increases the risk of CVD [37] and vascular damage disrupts frontal-subcortical-limbic neural pathways and connectivity in networks central to mood that predispose the development of depressive symptoms [38].

The interaction between AD, CVD, and depression is poorly understood. The aim of our study was to investigate the relationship between past depression and WMH burden in those with positive (AD+) and negative (AD-) CSF biomarker profiles for AD. We also aimed to better understand how CSF AD pathology and WMH burden relate to the clinical phenotypes of MCI and/or MDD. We compared AD CSF biomarker status (positive versus negative) and WMH burden among three groups: MCI, MCI with remitted MDD (MDD+MCI), and remitted MDD alone (MDD).

## MATERIALS AND METHODS

### Participants

Participants for this study were a subset of those enrolled in the Prevention of Alzheimer’s dementia with Cognitive remediation plus Transcranial direct current stimulation in Mild cognitive impairment and Depression (PACt-MD) study (ClinicalTrials.gov Identifier: NCT02386670) with a diagnosis of MCI, remitted MDD, or both who completed a lumbar puncture [39]. This multi-site, interventional study assessed the efficacy of a combination of transcranial direct current stimulation and cognitive remediation in the prevention of dementia in two over-lapping high risk groups: older individuals with MCI, or a history of MDD. Extensive clinical and research assessments, including a comprehensive neuropsychological battery, characterized participants’ cognitive performance. Participants were invited to provide biomarkers, including 3T MRI, CSF, peripheral blood biomarkers, genetics, PET, and EEG.

Diagnoses of MCI and MDD were made in accordance with the Diagnostic Statistical Manual of Mental Disorders, 5^th^ edition (DSM-5) [39]. The PACt-MD study required either a recent (defined as a history of a major depressive episode ((MDE) within≤5 years) or remote history of MDD in current remission. Evidence of the need for medical attention to confirm clinical significance of a remote MDE was required, and to provide support that DSM-5 diagnostic criteria were met. A geriatric psychiatrist performed a comprehensive assessment, including clinical neuropsychological testing and a score of≤10 on the Montgomery-Asberg Depression Rating Scale. The Structured Clinical Interview for the Diagnostic and Statistical Manual of Mental Disorders, 4^th^ edition confirmed the MDD diagnosis and ruled out the presence of excluded psychiatric disorders [39]. A comprehensive medical review ruled out the presence of physical conditions contributing to MCI. Our sample consisted of exclusively those with EOD that recurred (i.e., LLD). Patients with a clinical diagnosis of MCI (both amnestic and non-amnestic), remitted MDD, or both based on this clinical assessment were referred to the study and invited to sign the informed consent form approved by the CAMH Research Ethics Board.

### Assessment of WMH Burden

All participants underwent MRI scanning on the same 3-T GE MR750 Echospeed (General Electric, Milwaukee, WI) research-dedicated scanner at CAMH [39]. The fluid-attenuated inversion recovery (FLAIR) sequence was optional and was only obtained in a subset of the participants. Therefore, a trained neurologist (MCT) classified WMH burden as none/mild, moderate, or severe based on visual inspection of T2-weighted images.

### CSF collection and analysis

Approximately 15 mL of CSF was collected from each participant by allowing CSF to drip into a polypropylene collecting tube (gravity drip). The collecting tubes were then aliquoted and stored at –80°C within 30 min of collection. A sandwich ELISA was used to measure concentrations of Aβ_42_ (Innotest β-amyloid (1–42), Fujirebio), phosphorylated-tau (Innotest phospho-tau (181p), Fujirebio), and total-tau (Innotest hTAU-Ag, Fujirebio) following the manufacturer’s instruction [18, 19]. All samples were measured in duplicate and repeated if the difference between individual optic density values was greater than 20%. In addition to the ready-to-use calibrators (CAL) and Run Validation Controls (RVC) which were part of the Fujirebio Innotest assay kits, internal controls were also included in each run. After calculating the mean absorbance for the CAL, RVC, and unknown CSF samples, a sigmoidal 4-parameter curve fitting was used to determine the corresponding concentrations. CSF biomarkers were considered consistent with AD diagnosis (AD+) if phosphorylated tau > 68 pg/ml and Aβ_42_ to total tau index (ATI) < 0.8 [18, 19].

### Data analysis

Based on the results of the comprehensive neuropsychological assessments [39], composite scores were created for six cognitive domains: verbal memory, visuospatial memory, information processing speed, working memory, language, and executive function, in addition to an overall composite score [39]. First, AD+ and AD- participants were compared using *t*-tests for neuropsychological performance and Fisher’s exact test for WMH burden. The MDD group was excluded for the analysis of neuropsychological performance. Next, we compared diagnostic categories (MCI, MDD, MDD+MCI) using one-way ANOVA for CSF AD biomarkers and WMH burden. An alpha level of 0.05 was set to determine statistical significance. Statistical analysis was conducted using SPSS software (IBM SPSS 28). Given that the analyses were exploratory, no corrections were made for multiple comparisons.

## RESULTS

### Participants

We obtained CSF from 31 participants of whom 27 completed an MRI. Table 1 provides demographic information. Caucasian individuals were over-represented and, overall, participants were well-educated. Age, sex, self-reported race/ethnicity, or education did not differ among the three diagnostic groups.

**Table 1:** Demographic Characteristics and Diagnosis of the Sample

	Sample	MCI	MDD	MDD+MCI	Statistical Test
	*N* = 31	(*n* = 12)	(*n* = 7)	(*n* = 12)
Age, Mean (SD)	70.6 (4.40)	71.0 (4.6)	71.0 (4.9)	69.9 (4.2)	F(2,28) = 0.212, *p* = 0.81
Self-reported sex	20F:11M	7F:5M	5F:2M	8F:4M	Fisher’s Exact *p* = 0.90
Self-reported race (white/other)	23/8	8/4	7/0	8/4	Fisher’s Exact *p* = 0.28
Education (y)	15.3 (2.97)	15.3 (2.3)	16.4 (2.6)	15.3 (3.0)	F(2,28) = 0.697, *p* = 0.51
APOE ɛ4 (%)	41.9	58.3	14.3	41.7	*χ*^2^, *p* = 0.172
Anti-depressant Use (%)	38.7	0	71.4	58.3	χ^2^, *p* = 0.002^†^

MCI, mild cognitive impairment; MDD, major depressive disorder; F, female; M, male. ^†^statistical significance at the 0.05 level.

### CSF AD biomarkers and cognitive performance

Table 2 summarizes the cognitive performance in AD+ and AD- participants: AD+ participants performed significantly worse than AD- participants in verbal memory (*p* = 0.009).

**Table 2:** Demographic Characteristics, CSF AD Biomarkers, Cognitive Performance, and Burden of WMH among Participants with and without CSF Biomarkers Consistent with Alzheimer’s Disease Dementia (AD+ versus AD-*)

	AD+	AD–	Statistical Test	Cohen’s d
*N*	9	15
Age	70.9 (3.44)	70.2 (4.95)	t(22) = –0.368, *p* = 0.716	0.155
Self-reported sex	5F:4M	10F:5M	Fisher’s Exact, *p* = 0.678	–
Self-reported race (white/other)	6/3	17/5	*χ*^2^, *p* = 0.582	–
Education	14.6 (2.13)	15.3 (3.51)	t(22) = 0.547, *p* = 0.590	0.231
APOE ɛ4 (%)	66.7	27.3	*χ*^2^, *p* = 0.035^†^	–
Aβ (pg/mL), Mean (SD)	1347.2 (694.1)	691.3 (126.8)	t(22) = 2.783, *p* = 0.005	1.174
Total tau (pg/mL), Mean (SD)	208.2 (91.4)	705.0 (256.3)	t(22) = –6.894, *p* = <0.001	2.907
P-tau (pg/mL), Mean (SD)	89.8 (19.1)	47.2 (12.3)	t(22) = –6.523, *p* = <0.001	2.750
ATI, Mean (SD)	0.690 (0.232)	2.78 (1.19)	t(22) = 5.168, *p* = <0.001	2.179
Depression (Presence: Absence)	2 : 7	17 : 5	Fisher’s Exact, *p* = 0.012^†^	–
Overall Composite	–0.917 (0.698)	–0.330 (0.492)	t(22) = 1.782, *p* = 0.088	0.752
Verbal Memory	–1.82 (1.50)^†^	–0.391 (0.906) [*N* = 14]^†^	t(21) = 2.862, *p* = 0.009^†^	1.223
Visuospatial Memory	–0.777 (1.54)	–0.293 (0.780)	t(22) = 1.024, *p* = 0.317	1.179
Processing Speed	–0.674 (1.16)	–1.13 (1.12)	t(22) = –0.946, *p* = 0.354	0.399
Working Memory	–0.888 (0.763)	–0.447 (0.820)	t(22) = 1.306, *p* = 0.205	0.551
Language	–0.826 (1.02)	–0.248 (0.667)	t(22) = 0.739, *p* = 0.468	0.711
Executive	–0.514 (0.848)	–0.304 (0.553)	t(22) = 1.212, *p* = 0.24	0.312
No or Mild WMH	1 (12.5%)	0 (0)	Fisher’s Exact *p* = 0.031^†^	–
Moderate WMH	6 (75%)	8 (42.1%)
Severe WMH	1 (12.5%)	11 (57.9%)

ATI calculated as Aβ/(240 + 1.18×Tau). AD+ is defined as CSF phosphorylated tau > 68 pg/ml and Aβ_42_ to total tau index (ATI) < 0.8. ^†^Denotes statistical significance at the 0.05 level. CSF, cerebrospinal fluid

### CSF AD biomarkers and diagnosis

None of the 7 participants (0%) with a diagnosis of remitted MDD were AD+; 7 of the 12 participants (58.3%) with MCI were AD+; and 2 of the 12 participants (16.7%) with MDD+MCI were AD+ (*p* = 0.013). Participants with MCI exhibited greater levels of phosphorylated tau (P tau) than participants with MDD (*p* = 0.03) (Table 3).

**Table 3:** CSF AD Biomarkers and Burden of WMH in participants with MCI, MDD, or MCI+MDD

	MCI	MDD	MCI+MDD	Statistical Test
N	12	7	12
Aβ (pg/mL), Mean (SD)	963.1 (717.3)	1311.7 (275.6)	1239.4 (537.3)	F(28,2) = 1.052, *p* = 0.363
Total tau (pg/mL), Mean (SD)	473.0 (302.1)	173.8 (80.7)	315.9 (282.8)	F(28,2) = 3.001, *p* = 0.066
P-tau (pg/mL), Mean (SD)	71.1 (28.3) ^†^	40.6 (10.2)^†^	55.3 (21.8)	F(28,2) = 4.077, *p* = 0.028^†^
ATI, Mean (SD)	1.61 (1.61)	2.99 (0.54)	2.37 (1.07)	F(28,2) = 2.876, *p* = 0.073
AD Status (Positive: Negative)	7 : 5	0 : 7	2 : 10	Chi-Square, *p* = 0.013^†^
No or Mild WMH	1 [*N* = 11] (12.5%)	0 [*N* = 5] (0)	0 [*N* = 11]	Fisher’s Exact, *p* = 0.347
Moderate WMH	6 [*N* = 11] (75%)	1 [*N* = 5] (42.1%)	7 [*N* = 11]
Severe WMH	4 [*N* = 11] (12.5%)	4 [*N* = 5] (57.9%)	4 [*N* = 11]

ATI calculated as Aβ/(240 + 1.18×Tau). AD+ is defined as CSF phosphorylated tau >68 pg/ml and Aβ_42_ to total tau index (ATI)  <0.8. ^†^Denotes statistical significance at the 0.05 level. Post-hoc Bonferroni. CSF, cerebrospinal fluid

### CSF AD biomarkers and WMH

Table 3 presents the WMH ratings in AD+ and AD- participants: 26 of 27 participants who completed an MRI (96%) had moderate or severe WMH; AD- participants had more severe WMH burden than AD+ (*p* = 0.031).

## DISCUSSION

Our study examined the relationship between AD CSF biomarkers (AD+ versus AD-), diagnosis (MCI, MDD, or MDD+MCI), WMH burden and neuropsychological performance. Among the MCI participants in our sample, more than half (7/12) were AD+, while only 2/12 of those with MDD+MCI were AD+. As expected, all participants with AD+ CSF were cognitively impaired and performed worse than the AD- participants in verbal memory. In AD- participants, we did not observe a distinct cognitive profile. Finally, nearly all participants had moderate or severe WMH burden, irrespective of their diagnosis or AD biomarker status; however, AD- participants had more severe WMH burden.

Depression has been identified as one of twelve modifiable risk factors for dementia [1], and there is evidence to support a direct link to AD pathogenesis [15–23]. The neuropathological processes associated with MDD and increased dementia risk are highly variable [39, 40]. Age of onset of first episode and severity and duration of depressive episodes serve as important mediators for dementia and AD risk [42–44]. As discussed above, older patients with MCI or a history of recent or remote MDD which has recurred (i.e., LLD) are considered to be at high risk for cognitive decline. There is evidence that LLD is a prodromal state of neurodegenerative disease [45–47]. The PACt-MD sample attempts to capture those with remitted EOD and LOD. Among the 19 LLD participants in our sample, all had EOD; 12 had MCI, with CSF AD pathology evident in only two participants. These results suggest that the etiology of cognitive impairment among participants with a history of MDD may be distinct from participants with MCI alone [6]. This may also differ between patients with remitted versus non-remitted LLD; since our study excluded patients with current MDE, we cannot comment on this group.

The role of WMH in cognitive decline and the etiology of dementia is well established [48, 49]. Nearly all participants in our sample (26/27) exhibited moderate or severe WMH, however a sizeable number (*n* = 7) of participants were cognitively intact despite significant CVD burden. Participants with AD+ CSF performed worse in verbal memory than AD- participants, consistent with the neurocognitive profile of AD [50, 51]. All participants with MCI exhibited slowed processing speed, which is typical of vascular cognitive impairment in the context of moderate to high WMH burden [52]. Variations in the spatial distribution of WMH are known to contribute to a range of clinical phenotypes [53]. Although our study did not assess WMH localization, periventricular WMH are more closely related to cognitive impairment than deep WMH [28–30]; frontal [31–34] and temporal [35] WMH are associated with depression. We hypothesize that vascular lesions in clinically meaningful loci may account for those exhibiting MCI, given that only 38% of those with MCI had positive CSF AD biomarkers (*n* = 9). The sizeable WMH burden in our sample suggest that protective processes such as cognitive reserve [62] may be contributing to intact cognition in the MDD group. Participants in our sample were highly educated, with a mean of 15.3 years of education, lending support to this hypothesis.

The ɛ4 allele of the apolipoprotein E (APOE4) is a well-established risk factor for AD [63]. In our sample, the frequency of APOE4 was as expected significantly higher among AD+ participants. The relationship between APOE and AD is poorly understood. One theory suggests that APOE4 may impair the clearance of Aβ, contributing to its accumulation [63]. There is mixed evidence that APOE4 carrier status may increase the risk of depression [64] and influence the association between WMH and cognitive decline in AD [65]. In our sample, the MDD+MCI group has a greater proportion of APOE4 carriers compared to the MDD group (41.7% versus 14.3%, respectively), however, our small sample size precludes any analysis of the relationship between APOE4 status, depression, WMH, and AD.

Study limitations include the limited generalizability of our results arising from the small sample size, and a predominantly White and highly educated demographic. We did not capture potentially salient details of prior MDEs, such as episode frequency, which could have potential correlations to WMH burden and an evolving vascular neurocognitive disorder. Anti-depressant use also appears to mediate improved cognitive performance among individuals with depression [66]. Anti-depressant use appeared to be more common in the MDD group compared to the MDD+MCI group (71.3% versus 58.3%, respectively); however, we were unable to perform statistical analyses to control for anti-depressant use due to our small sample size. We may also have underestimated WMH burden using non-fluid attenuated T2 images rather than FLAIR images, which is the gold standard for WMH quantification. Finally, despite the use of well-validated diagnostic criteria of the DSM-5, diagnosing a remote MDE remains unreliable [39, 67].

Further research with larger samples sizes are needed to examine the potential contribution of WMH in patients with a remitted MDD and MCI in conjunction with pathological biomarkers for AD and CVD. A causal link between WMH burden and depression with associated persistent cognitive impairment would provide a rationale for primary prevention of depression with control of vascular risk factors.

## Data Availability

The data supporting the findings of this study are available on reasonable request from the corresponding author.
